# Proterozoic microfossils continue to provide new insights into the rise of complex eukaryotic life

**DOI:** 10.1098/rsos.240154

**Published:** 2024-08-21

**Authors:** Ross P. Anderson, Sanaa Mughal, George O. Wedlake

**Affiliations:** ^1^ Museum of Natural History, University of Oxford, Oxford OX1 3PW, UK; ^2^ All Souls College, University of Oxford, Oxford OX1 4AL, UK; ^3^ Department of Earth and Atmospheric Sciences, University of Alberta, Edmonton, Alberta T6G 2E3, Canada; ^4^ Department of Earth Sciences, University of Oxford, Oxford OX1 3AN, UK

**Keywords:** complex life, eukaryotes, organic-walled microfossils, Proterozoic Eon

## Abstract

Eukaryotes have evolved to dominate the biosphere today, accounting for most documented living species and the vast majority of the Earth’s biomass. Consequently, understanding how these biologically complex organisms initially diversified in the Proterozoic Eon over 539 million years ago is a foundational question in evolutionary biology. Over the last 70 years, palaeontologists have sought to document the rise of eukaryotes with fossil evidence. However, the delicate and microscopic nature of their sub-cellular features affords early eukaryotes diminished preservation potential. Chemical biomarker signatures of eukaryotes and the genetics of living eukaryotes have emerged as complementary tools for reconstructing eukaryote ancestry. In this review, we argue that exceptionally preserved Proterozoic microfossils are critical to interpreting these complementary tools, providing crucial calibrations to molecular clocks and testing hypotheses of palaeoecology. We highlight recent research on their preservation and biomolecular composition that offers new ways to enhance their utility.

## 1. Introduction

Examine the modern biosphere, and you’ll find a world teeming with eukaryotic life. Eukaryotes are those organisms whose cellular machinery is compartmentalized into membrane-bound organelles like the nucleus and mitochondrion, and they are the only organisms to have achieved complex multicellularity [1]. Today, eukaryotes comprise >80% of all documented species on the Earth [2] as well as >80% of our planet’s biomass [3]. They also account for most net primary productivity [4].

**Figure 1.**
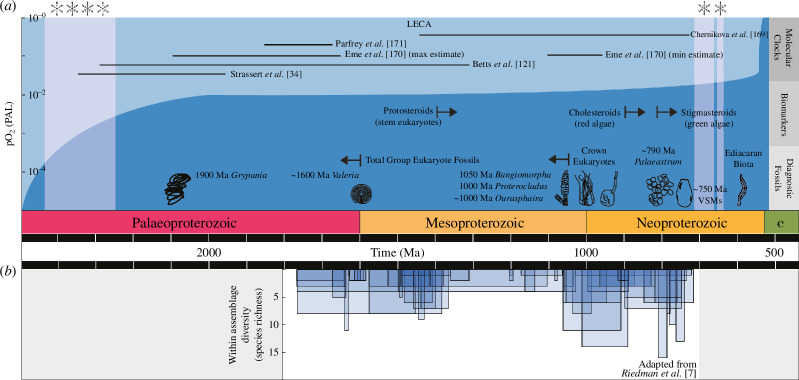
Summary of eukaryote evolution across the Proterozoic Eon and Cambrian Period set against palaeoenvironmental change. The level of atmospheric oxygen is shown in blue [5] with periods of widespread glaciation also indicated [6]. (*a*) Molecular clock, biomarker and fossil record evidence for the rise of eukaryotes is plotted, along with (*b*) within-assemblage diversity from [7].

The initial diversification of eukaryotes occurred across the Proterozoic Eon (2500−539 million years ago, Ma) and had profound consequences for our planet, its biogeochemical cycles, as well as its incumbent prokaryotic (Bacteria and Archaea) life, setting the stage for the foundation of the modern biosphere and Earth system (figure 1) [8–10]. The emergence of eukaryotes as primary producers, for example, may have transformed the oceanic biological pump, facilitating greater organic carbon export to depth and expanding oxygenated surface waters downward [11–13] (but see [14]). Additionally, the evolutionary innovations of eukaryotes such as sexual reproduction [15] and predation [16] altered ecosystems, evolutionary dynamics and biogeochemistry [17,18]. Biomineralization was a particularly key eukaryotic innovation [19,20]. The evolution of skeletons in early animals, for example, helped drive a predator–prey arms race [21], and the acquisition of siliceous skeletons in protists (initially in radiolarians) fundamentally altered the budget of dissolved silica in the oceans [22], with possible wide-ranging effects on the production of authigenic clays, the carbon cycle and global climate [23,24].

Given the central importance of eukaryotes to the history of life and environments on the Earth, it is surprising that our understanding of their initial diversification and proliferation, its evolutionary tempo and triggers, remains limited. Although we know the broad time interval over which the rise of eukaryotes occurred, we are yet to precisely constrain the timings of major events such as the evolution of the crown clade or the evolution of major groups within the crown (e.g. green and red algae, fungi and animals) [8,10]. Neither do we have a good understanding of the ecology of these earliest eukaryotes; we have learnt much about Proterozoic environments over the last few decades [5,25–27], but we still do not know the range of palaeoenvironments that eukaryotes inhabited, nor how it may have shifted through time.

The central challenge has been the rarity of fossil evidence with detailed geological contexts. The earliest eukaryotes were commonly microscopic and lacked the mineralized shells and skeletons that characterize most fossil organisms [20,28,29]; consequently their fossils are poorly documented when compared with those of their Phanerozoic relatives [30]. Moreover, variations in the quality of preservation between different fossil assemblages [31] mean that fossils with enough morphological features to be assigned to specific eukaryotic clades are rarer still [8]. Despite these limitations, the Proterozoic record of fossil eukaryotes has grown significantly over the last 70 years and provides the only direct means to view early eukaryotes, their biology and ecology [8–10,30,32,33].

In this article, we review what is known about the rise of eukaryotes from fossil evidence, specifically organic-walled microfossils and how the rarity of these fossils has encouraged the use of complementary lines of evidence to reconstruct ancestry, principally molecular clocks [34] and geological biomarkers of sedimentary organic matter [35]. We argue that the Proterozoic microfossil record remains vital to efforts to accurately reconstruct the tempo of the evolution of the earliest eukaryotes and their ecologies and highlight its importance for interpreting the complementary lines of evidence. We show how new tools are unlocking and renewing its rich potential as a source of evolutionary information.

## 2. The status quo

### 2.1. Preserving early eukaryotes as fossils

The study of Proterozoic organic-walled microfossils dates back to the 1950s when discoveries were made in parallel in early diagenetic marine cherts of Ontario, Canada (the famed Gunflint Chert) [36] and marine mudstones of Russia [37]. Over the subsequent ~70 years, marine cherts [38,39] and mudstones [40,41] have emerged as the primary repositories [30], with early diagenetic phosphates [42–44] and carbonates [45,46] also gaining importance. Beyond the marine realm, some microfossils may derive from mudstones and phosphates deposited in lacustrine or estuarine settings [44,47,48].

Fossilization processes for organic-walled microfossils are somewhat well understood in Proterozoic cherts [49] and phosphates [50] where fossils are preserved in three dimensions. This understanding of the underlying fossilization processes in these lithologies allows us to recognize preservation bias in their microfossil records. Marine chert deposition was favoured in the Proterozoic, in comparison to today, due to elevated marine dissolved silica concentrations before skeletal organisms sequestered silica (sponges, radiolarians and diatoms all subsequently evolved siliceous skeletons) [22,24,51]. However, chert commonly only fossilized the shallowest Proterozoic communities in peritidal settings where silica saturation was higher than in deeper waters due to enhanced evaporation [51]. Ecology may have also played a role: enhanced run-off from continental environments provided nutrient resources for coastal communities, leading to greater concentrations of organic matter in these settings, which provided abundant nuclei for chert precipitation [49,52]. Experimental data show how the biopolymers associated with different marine organisms may have affected the propensity for silica to precipitate on organic substrates [53,54], potentially imparting phylogenetic bias on the microfossil record. Fossilization of cellular materials in phosphate-rich sediments [55] and diagenetic phosphate nodules [44] was controlled by complex interactions between redox and phosphatization [50]. It was often favoured in shallow sub-tidal [56], or possibly lacustrine settings [44], near the suboxic/anoxic sediment boundary below oxic bottom waters; only organisms either living in these settings or transported post-mortem to them were susceptible to phosphatization [56].

The processes of fossilization are less well understood for Proterozoic microfossils preserved as compressions in marine and lacustrine/estuarine mudstones [31], which is particularly concerning as they host >75% of Proterozoic eukaryote-bearing assemblages [30]. Preservation may have been comparable to that of decay-resistant palynological material and/or small carbonaceous remains of early animals from the Phanerozoic [57]. Palynological material can be recovered from Phanerozoic mudstones that range in their levels of total organic carbon (TOC; e.g. [58]). However, the level of TOC has recently been shown to exert a primary control on the presence/absence of microfossils in Proterozoic mudstones as well as on the quality of their preservation, with microfossils usually confined to rocks with <0.5 weight percent (wt%) TOC (mean TOC of rocks with fossils = 0.32 wt%, mean TOC of rocks without fossils = 0.72 wt%) [57,59]. Woltz *et al*. have explained this phenomenon, arguing populations of heterotrophic bacteria, which degrade microfossils, cannot sustain themselves if disseminated organic carbon (i.e. TOC) is not available in high enough quantities or they have to forage for it across large distances through sedimentary pore space [57,59]. Commensurate with this idea, higher clay content in sediments has also been shown to be an important factor by reducing permeability and increasing the chances that bacterial routes to carbon food sources are impeded [57,60,61].

A growing body of evidence suggests not all microfossils preserved in Proterozoic mudstones are created equal, with some perhaps evidencing markedly higher quality preservation, enabling more biological information to be gleaned from them [31]. Microfossils in select deposits, for example, the Tonian Svanbergfjellet Formation (Svalbard, Norway), Wynniatt Formation (Canada) and Lakhanda Group (Russia), may show fine morphological details including multicellularity and fine spines/processes [31,62]; the microfossils can even have cell walls as little as tens of nanometres thick [62]. Nearly 30 years ago, Butterfield compared this enhanced preservation with Burgess Shale-type (BST) preservation of early soft-bodied animals in Cambrian mudstone deposits [31]. A myriad of factors has been proposed to contribute to BST preservation from limited oxidant supply to sedimentary sealing through authigenic carbonate [63–67], with recent work focusing on the roles played by clay minerals. Both the iron-rich clay berthierine and the aluminium-rich clay kaolinite have been shown to be toxic to heterotrophic decay bacteria [68], with kaolinite also increasing the decay resistance of organics by facilitating their polymerization [69]. Cambrian deposits with BST fossils are rich in berthierine [70], and kaolinite has been shown to template tissues of fossils from the Burgess Shale (Canada) [64,71]. Geochemical data suggest microfossils from Proterozoic deposits with enhanced preservation are spatially adjacent to enrichments of kaolinite, with the mineral potentially serving as a protective halo to fossil cell walls, promoting comparisons with Cambrian BST preservation [62,72,73].

Given the similarities to BST preservation, this enhanced type of preservation in mudstones may bias our primary record of early eukaryotes towards environments or times when clay deposition was favoured. Kaolinite production, for example, is commonly favoured in tropical environments with high drainage and a low soil pH [74]. Congruent with a tropical locus for clay production, the three example deposits with enhanced preservation, identified above, sit in the palaeotropics or mid-latitudes [75]. Enhanced preservation may also have been favoured in the Proterozoic when authigenic clay production is inferred to have been higher than today [23,24,76,77]. Could periods of enhanced weathering, for example, in the immediate aftermath of Cryogenian Snowball Earth glaciations [6,78,79], have facilitated enhanced preservation?

Beyond the enhanced record, compilations of TOC from Proterozoic mudstones identify the Neoproterozoic as an interval of unusually low TOC in the context of the Earth’s history, making it perhaps particularly conducive to the preservation of organic-walled microfossils more broadly [59,80]; indeed, the Neoproterozoic hosts assemblages with high recorded biodiversity [30].

### 2.2. Recognizing early fossil eukaryotes and their diversity

With a growing global record of Proterozoic microfossils in multiple lithologies, how can we tell which ones represent fossilized remains of eukaryotes rather than bacteria or archaea? Answering this question has proved a challenge of recognition in the face of few distinguishing characters, and the reliance on those characters being preserved [8,10,81]. In contrast to prokaryotic cells, eukaryotic cells possess features including a nucleus, complex cytoskeleton, sophisticated endomembrane system and mitochondria [81]. The preservation potential of many of these sub-cellular level characters remains doubtful, even in exceptionally preserved material, rendering their recognition in Proterozoic microfossils challenging [82,83]. Complicating matters further, a number of these distinguishing characteristics were acquired between the first common ancestor of eukaryotes (FECA) and their last common ancestor (LECA), i.e. between the origin of total group eukaryotes and crown group eukaryotes [81]. Unravelling their presence or absence in Proterozoic microfossils is critical to determining the sequence of character acquisition in the eukaryote crown group lineage [81].

**Figure 2.**
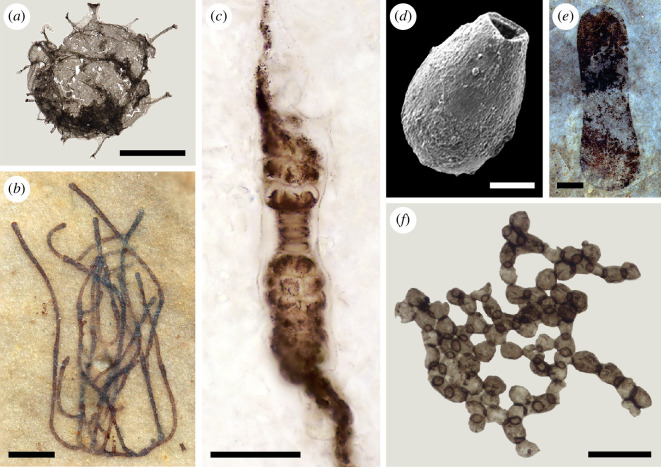
Examples of key Proterozoic fossils that represent probable eukaryotes. (*a*) *Tappania plana*, a total group eukaryote from the Greyson Formation, Belt Supergroup, Montana, USA, image courtesy Adam [87]. (*b*) *Proterocladus antiquus*, a total group green alga from the Nanfen Formation, Xihe Group, North China, image courtesy Tang [88]. (*c*) *Bangiomorpha pubescens*, a total group red alga from the Hunting Formation, Somerset Island, Nunavut, Canada, image courtesy Butterfield [8]. (*d*) *Trigonocyrillium horodyskii*, a vase-shaped microfossil (VSM) from the Kwagunt Formation, Chuar Group, Arizona, USA, image courtesy Porter [89]. (*e*) *Tawuia dalensis*, a possible macroalga from the Shiwangzhuang Formation, Tumen Group, North China, image courtesy Tang [90]. (*f*) *Palaeastrum dyptocranum*, a possible total group green alga from the Svanbergfjellet Formation, Akademikerbreen Group, Svalbard. Scale bars, (*a*,*c*,*f*) 50 µm, (*b*) 200 µm, (*d*) 25 µm, and (*e*) 1 mm.

Over the last few decades, a suite of characters has emerged which, when used in combination, determines whether single-celled Proterozoic microfossils represent eukaryotes at the precision of identifying the total group. These are large size (>100 μm maximum cell dimension), possession of complex spines or processes extending from the cell wall and/or cell wall ornamentation, and cell walls with complex ultrastructure [32,84–86]. However, the last of these commonly requires advanced imaging techniques such as transmission electron microscopy (TEM), which are technically challenging and may not be readily available [85]. Globally, rocks ~1650 Ma and younger harbour microfossils with this combination of characters, providing a minimum estimate for total group eukaryote antiquity (figure 1*a* and 2) [7,86,87,91–93].

However, older fossils exist with claims on eukaryote affinity; for example, fossils of vesicles from the Archean Moodies Group, ~3200 Ma in South Africa, exhibit large size and may represent single eukaryotic cells [94]. Several bacterial groups have also achieved large size [95,96], and eukaryotes can be as small as 1 μm in modern oceans [97], rendering size alone an unhelpful character for determining eukaryotic affinity. The microfossil *Navifusa*, which can be up to 550 μm long, represents a cautionary tale; despite its large size, it was recently suggested to be a potential cyanobacterium based on the documentation of possible thylakoid membranes [98]. Macroscopic pyritized structures from the Gabon ~2100 Ma [99–101], carbonaceous forms such as *Grypania* ~1900 Ma [102] and *Katnia* ~1600 Ma [103] and decimetre-scale blade-like carbonaceous compressions [104,105] have also all been allied to eukaryotes but without conclusive evidence; other origins either as bacteria or as non-biogenic structures exist [8,84,106].

More recently, Porter [81] and Porter & Riedman [10] have delved deeper into the confirmed record of Palaeoproterozoic eukaryote microfossils, building on earlier papers that asked what these earliest fossilized eukaryotes can tell us about the acquisition of eukaryotic characters [84,107,108]. The disparity of processes extending from the cells of the microfossil *Tappania plana* (figure 2*a*), for example, provides evidence for the evolution of eukaryotes’ dynamic cytoskeleton, which allowed them to shape their cells in response to environmental stimuli [87,107]. Encystment, common among diverse eukaryotes today [109], is also evidenced in Palaeoproterozoic microfossils through medial splits [91,110] and pylomes [7,91,111–113], although some of these may be the result of post-depositional compaction and/or breakage and degradation [10]. *Valeria lophostriata* has evenly spaced concentric ridges on the inner surface of its vesicle with circular foci at opposite poles [114], which may indicate that the organism possessed a mechanism to determinately rupture its cyst [115]. Other fossils, e.g. *Satka favosa*, are constructed of repeated units or plates that argue for the presence of intracellular vesicles, intracellular trafficking and exocytosis in Palaeoproterozoic eukaryotes [84,116]; these plates likely formed individually [10] as in some specimens they do not fit perfectly together [116], and today units are created individually in the golgi apparatus of eukaryote cells before being joined [10]. Such plates may also suggest the earliest fossilized eukaryotes had the ability for phagocytosis [10,117].

None of the microfossils so far discussed, however, is an unequivocal representative of crown group eukaryotes, which share the common ancestor LECA. It is possible that some are crown eukaryotes, but they do not possess enough preserved morphological characters to be identified [8,10,81]. Unequivocal crown eukaryotes only emerge in the record from ~1050 Ma, with fossils that can be associated, although not always as precisely as we would like, with modern eukaryotic groups [8]. The classic example, which emphasizes the challenges of accurate phylogenetic placement for even the best preserved microfossils, is *Bangiomorpha pubescens* [15,118], ~1050 Ma from Arctic Canada [119]. This multicellular form with distinctive patterns of radial wedge-shaped cells in multiseriate filaments (figure 2*c*) has been allied to modern crown red algae (Rhodophyta) within the family Bangiaceae [15]. However, its cell pattern is no longer thought to be exclusive to Bangiaceae and may be present across a range of basal rhodophyte groups [120], meaning a more cautious approach to its affinity at the level of total group rhodophytes is necessary [121]. Other confirmed early crown eukaryotes from rocks older than the Ediacaran include the ~1000 Ma *Proterocladus antiquus* (figure 2*b*) [88] and ~790−730 Ma vase-shaped microfossils (VSMs) [89,122–124]. *Proterocladus antiquus* may be a crown member of Cladophorales, although more cautiously it at least represents total group green algae and likely total group Chlorophyta [88,125]. VSMs (figure 2*d*) are thought to be at least total group amoebozoans [89,122–124]. Numerous other pre-Ediacaran microfossils, some as old as ~2400 Ma, have been argued to represent crown eukaryotes but with varying degrees of phylogenetic support, e.g. possible algae [83,126,127], euglenids [48], fungi [128,129], holozoans [47], sponges [130] and stramenopiles [131]. New approaches such as quantitative morphological analysis and biomolecular characterization, both discussed later in this article, may yield further insight to how we might confidently resolve the placement of these phylogenetically ambiguous microfossils.

Beyond the microfossil record, macroscopic carbonaceous remains have also been reported from Proterozoic strata [132,133]. For example, specimens of *P. antiquus* can be macroscopic in size [88]. However, the generic overall morphology of most of these macroscopic fossil taxa may mask their polyphyletic nature [134], with individual specimens representing eukaryotic organisms, possibly macroalgae [135], while others are more likely colonial bacteria [136]. Among the most interesting macroscopic fossils are the sausage-shaped *Tawuia* (figure 2*e*) and *Archaeochaeta*, both of which present cases for being macroalgae [135,137].

### 2.3. Fossil record views on Proterozoic eukaryote evolution

What can these Proterozoic microfossils tell us about the tempo and ecology of the early evolution of eukaryotes? At the most basic level, we can use them to ask when the ancestor of modern eukaryotes evolved (figure 1). As already noted, Porter [81] and Porter & Riedman [10] have looked at the timing of the evolution of LECA in some detail. While the fossil record confirms the existence of total group eukaryotes by ~1650 Ma [7,86,87,91–93], at present the origin of crown eukaryotes and evolution of LECA are relatively poorly constrained. *Bangiomorpha pubescens* and *P. antiquus* place a minimum age of ~1050 Ma on crown eukaryotes but whether the crown clade evolved closer to 1650 or 1050 Ma remains to be determined: the early versus late LECA hypotheses [8,10,81,127]. Fossils in the intervening interval possess some but not all defining characters of crown eukaryotes, the major missing character being indications of the ability for sterol biosynthesis [10,81]. The recent discovery of possible photosynthetic eukaryotes in rocks ~1630 Ma, however, may lend support to the early LECA hypothesis [127].

Total group eukaryotes may have initially diversified in the Palaeoproterozoic, as suggested by fossil reports and models based on fossil sizes [7,138,139]. However, the microfossil record indicates a diversification of major crown eukaryote clades from the late Mesoproterozoic through the Tonian [8,140]. The appearance of *B. pubescens* and *P. antiquus* as total group representatives of red and green algae, respectively, indicates that Archaeplastida had diverged at a minimum by ~1050 Ma [15,88,119], although new fossils may suggest it did so as far back as ~1630 Ma [127]. VSMs show that by ~790 Ma total group Amoebozoa was also established [89,122,123]. Beyond these constraints, however, determination of when the major groupings of eukaryotes evolved remains elusive until the emergence of metazoan fossils in the Ediacaran [141–146].

A Tonian diversification of eukaryotes more broadly is evidenced in compilations of organic-walled microfossils with uncertain but likely eukaryotic affinity. Riedman and Sadler show how form-species richness peaks at ~770 Ma, although the total numbers of eukaryotic taxa (<40) remain low in comparison to younger time intervals [147]. Within-assemblage diversity remains fairly constant throughout at <20 taxa (figure 1*b*)—might this indicate a limit to the complexity of individual Proterozoic ecosystems [7,30]? Following the Tonian, eukaryote diversity declines markedly through the ~720−635 Ma Cryogenian with its Snowball Earth global glaciations; most assemblages comprise only simple spheroidal fossils [147,148], although there are some assemblages that merit further investigation [46,149]. The Ediacaran witnesses the radiation of spiny acanthomorphic acritarchs, with form-species richness increasing dramatically, although the affinity of these taxa remains uncertain [150–153].

The Proterozoic microfossil record also documents ecological expansion, particularly with regards to feeding mode [16–18,83]. The late Mesoproterozoic-Tonian microfossil *Arctacellularia tetragonala* yields biomolecular evidence for chlorophyll residues, providing direct evidence of photosynthesis within eukaryotes [83]. In the Tonian, heterotrophic organisms are recognizable for the first time with the emergence of VSMs [89,122–124]. Further evidence for the proliferation of protistan predators can be found in the perforations in the cell walls of microfossils that extend to the latest Mesoproterozoic [16,154,155]; disparity in the shapes and sizes of perforations suggests a variety of predatory species [154] and perhaps selective predation [155]. Moreover, there is evidence of enhanced structural defences to predation with diverse microfossils from the Tonian Fifteenmile Group (Canada) recording controlled hydroxyapatite biomineralization of protective scales [19,29,156]. The evolution of more resistant bodies may not only have conferred an advantage to early eukaryotes in defence but also in attack [157]. In addition to antagonistic ecological relationships, the Proterozoic fossil record documents possible symbioses. For example, ectosymbionts have been reported in association with the macrofossils *Tawuia* and *Sinosabellidites* [90], with external sculpturing of other fossils perhaps indicative of their presence among Proterozoic eukaryotes more widely [10].

Finally, Proterozoic microfossils derive from geological contexts that provide the potential to establish the habitats of early eukaryotes; they may have inhabited these geological contexts or been transported to them post-mortem. The majority of documented deposits with Proterozoic microfossils are shallow marine [30], including those hosting the oldest fossil eukaryotes [7,86,87,91–93] and confirmed examples of crown eukaryotes [15,88,89,118,122,123]. However, a growing number of microfossil occurrences are also being recovered from non-marine deposits [44,48,158] argued to be lacustrine [44,48] or estuarine [159–162]. Beyond these gross determinations of depositional environment, a number of studies have attempted to understand how eukaryote diversity and abundance vary within a single setting, with perhaps the best example coming from Javaux & Knoll [116] building on earlier work [107,163]. They looked at the abundance and diversity of microfossils of likely eukaryotic affinity across a shelf–slope transition in the Mesoproterozoic Roper Group (Australia) showing that diversity was highest in shallow shelf environments. Given the challenges in preserving early eukaryotes, taphonomic bias must be mapped onto this pattern; basinal shales have higher TOC and thus lower preservation potential, possibly masking any true ecological signal [116]. Nevertheless, a similar onshore–offshore diversity trend has been reported for the Mesoproterozoic Bangemall Group (Australia) [164]. In cherts of the Tonian Draken Formation (Svalbard, Norway), likely eukaryotes are mostly restricted to lagoonal facies [38], whereas in phosphorites and cherts of the Doushantuo Formation (China), diversity within a variety of coastal environments has been reconstructed [56]. Koehler and colleagues provide an important example of the potential for such detailed palaeoecological data from within individual deposits to be combined with geochemical proxies to evaluate hypotheses for any environmental restriction of eukaryotes [165]. In the case of the Roper and Bangemall examples, they argue that access to nitrate may have limited the initial dispersal of eukaryotes among marine settings [165], although salinity may also have been important in marginal marine settings [162].

## 3. Complementary evidence and the need for Proterozoic microfossils

Despite the immense progress that has been made in the last ~70 years, much remains unknown about the early evolution of eukaryotes. Few Proterozoic microfossils preserve enough details to allow us to track the evolution of the eukaryotic cell or the emergence of major groups with the temporal precision that we would wish. Moreover, palaeoecological reconstructions are plagued by limited controls on non-preservation versus the true ecological absence of eukaryotes. In the absence of a comprehensive microfossil record, palaeontologists have turned to complementary lines of evidence to understand early eukaryote evolution, chiefly molecular clocks, biomarkers and geochemistry.

### 3.1. Molecular clocks

Molecular clocks rely on the genetic information encoded in modern organisms to create a phylogeny before using an evolutionary model to determine the ages of key nodes [166,167]. Over the last 20 years, the use of such methods has become widespread in order to determine the topology of the eukaryote tree, the timing of the origin of crown eukaryotes, as well as the tempo of their diversification into major clades [9,168]. However, such estimates from molecular clocks have wide uncertainties (figure 1*a*). For example, estimates for the age of LECA cover almost the entirety of the Meso- and Palaeoproterozoic: 718−1455 Ma [169], 1007−1898 Ma [170], 1210−1843 Ma [121], 1679−1866 Ma [171] and 1958−2386 Ma [34]. Such large ranges provide little clarity around the tempo of early eukaryote evolution and limit our ability to assess how it was linked to environmental change through accurate temporal correlations with geological and geochemical data.

Critical to efforts to improve the temporal precision of molecular clocks is the integration of fossil data as calibrations [172–174]. Fossils can be used as minimum temporal constraints, confirming that an individual clade had already evolved at a specific timepoint. When integrated in a Bayesian framework, these calibrations can be given soft bounds, i.e. probabilities that the constraints are violated by a poorly sampled fossil record [167,175]. However, currently, there are too few Proterozoic fossils that can be placed accurately into phylogeny to be used for these purposes. Betts and colleagues provide a summary of the constraints known in 2018 [121]. Of the crown eukaryote fossils we have discussed already, only *B. pubescens*, as a minimum bound on total group rhodophytes, was integrated in their analyses with the earliest eukaryote microfossils employed as a minimum constraint of 1619 Ma on total group eukaryotes [121]. Other molecular clocks have used *B. pubescens* to constrain more derived clades, e.g. bangiophytes, with choice of phylogenetic placement having a significant effect on estimates for the timescale of eukaryote evolution [81]. There is a pressing case for palaeontologists to provide more Proterozoic fossils, which sample eukaryote phylogeny broadly, to integrate into molecular clock models.

Molecular data not only provide insight into the timescale of eukaryotic evolution through molecular clocks but also enable probabilistic estimates of the biology and ecology of ancestral eukaryotes based on the characteristics of those living today. For example, so-called ancestral state reconstruction has been applied to investigate the ancestral habitats of eukaryotic algae, suggesting that both red and green algae likely evolved in freshwater environments before diversifying later, perhaps around the Tonian or Cryogenian, into the marine realm [125,176]. Other ancestral state reconstructions have looked at feeding modes, arguing that the emergence of eukaryovory in the Neoproterozoic may have spurred eukaryote diversification [17]. However, the accuracy of ancestral state reconstruction is dependent on the evolutionary model employed [177,178]. The hypotheses created by ancestral state reconstructions require testing with fossil data. Fossils placed in their geological context can test hypotheses of habitat occupation [38,107,116,163,164], and feeding mode can be inferred from microfossil morphology and biomolecular composition [16,18,83,154]. Do growing fossil occurrences from marginal marine settings, where salinity likely fluctuated [44,48,159], add weight to the reconstruction of freshwater habitats for ancestral algae [125,176]?

### 3.2. Biomarkers and other geochemical indicators of early eukaryotes

A second tool that has gained prominence in recent years is the documentation of organic biomarkers derived from preserved sedimentary organic matter. Specifically, steroids have been targeted [35,179–181], since these represent the geological product of sterols, a biomolecule known to be produced by eukaryotes [182]. Brocks and colleagues have produced the most comprehensive account of steroid biomarkers to date (figure 1*a*) [35,179]. They show the oldest steroids known to be made by crown group eukaryotes date to the Tonian, represented by saturated steranes, commonly cholestane which is a biomarker for red algae [35,179] (but see [183]). Following the Cryogenian Snowball Earth glaciations, Ediacaran biomarker abundances change, with a rising proportion of stigmasteroids characteristic of chlorophyte green algae [35,179]. Prior to the Tonian, aromatic protosteroids, possibly characteristic of stem eukaryotes, have been recovered as far back as the Palaeoproterozoic [35,179].

If correct, the biomarker record presents a view of early eukaryote evolution that is broadly in accordance with the microfossil record: stem eukaryotes evolved before the Palaeoproterozoic and proliferated in Proterozoic oceans, with crown eukaryotes originating much later just prior to the Tonian [35], although recent fossils may argue against such a late origin of crown eukaryotes [127] and little is known of the non-marine Proterozoic biomarker record.

The biomarker record also provides complementary data to the fossil record, not necessarily telling us exactly when clades originated, but rather when they proliferated and began to contribute a substantial component of sedimentary organic matter [9,35]. This is likely the case with the rise of chlorophyte algae, where the ~1000 Ma fossil *P. antiquus* argues for a much earlier origin of total group chlorophytes [88] before their proliferation in the Ediacaran evidenced by stigmasteroid biomarkers [35,179]. An increase in 2-methylhopanoids after 600 Ma has also been attributed, although indirectly, to the Ediacaran rise of eukaryotic algae via their mutualistic relationship with Alphaproteobacteria (a maker of the biomarker) [184]. Returning to the origin of LECA, the biomarker record does not rule out an origin earlier than the Tonian, it can only argue that, before then, crown eukaryotes were not contributing substantially to preserved organic matter [35].

Drawing definitive conclusions from biomarker studies, however, is not easy [9]. Further analysis of reported eukaryotic steranes from Archean rocks in Australia, hundreds of millions of years before the microfossil record of eukaryotes [185], determined them to be modern contaminants [186]. Moreover, as with the fossil record, the role of taphonomic bias in documented relative abundances of individual steroids remains to be fully determined; do microbial degraders preferentially degrade certain biomarkers over others [187–189]? The biomarker record is poorly preserved in oxygenated, high-energy and shallow water environments, often the environments captured by the fossil record; is the biomarker record not sampling these key habitats for early eukaryote evolution [35]? Finally, the utility of biomarkers can suffer from uncertainties in the phylogenetic distribution of biomolecules today [190–194].

Beyond biomarkers, the isotopic composition of sedimentary zinc is emerging as a signature for reconstructing early eukaryote evolution. Shifts in Zn isotopes in sulfides and organic materials ~800 Ma have been attributed to the proliferation of eukaryotes as they sequester more Zn biologically than bacteria and archaea [195]. However, as with biomarkers, Zn isotopic data provide only an indication that eukaryotes were contributing to biogeochemical cycles in a significant way, and may thus fail to capture early eukaryotes if they were rare.

## 4. Improving the utility of Proterozoic microfossils

**Figure 3.**
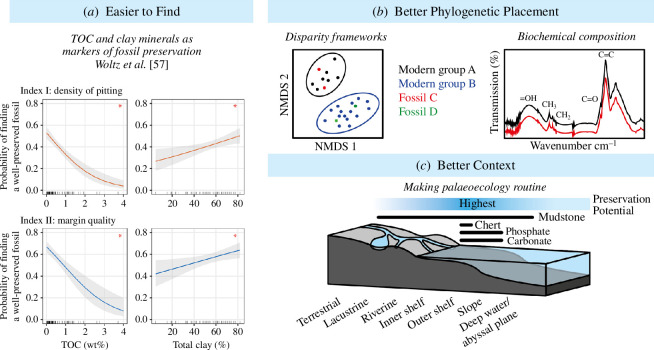
New avenues to improve the utility of the Proterozoic microfossil record. (*a*) Recent taphonomic work suggests well-preserved microfossils are found in rocks poor in TOC and rich in clay minerals [57]. (*b*) Schematic diagrams showing the utility of quantitative analyses of modern eukaryote morphological disparity and fossil biomolecule characterization for more accurate phylogenetic placement of fossils. (*c*) Schematic diagram of a shelf–slope transition showing preservation potential and highlighting the palaeoecological information that fossils harbour.

Integration of the fossil record with complementary molecular clocks and biomarkers will provide a fuller picture of early eukaryote evolution [9]. However, the Proterozoic microfossil record remains vitally important. It provides the only way to directly glimpse extinct organisms, providing evidence of morphological innovations and, through geological contexts, palaeoecology. It also validates the evolutionary hypotheses made by molecular clocks and biomarkers. Recent research is tackling how the utility of the microfossil record can be further improved (figure 3), addressing how we might (i) more easily find biologically informative microfossils, (ii) better assess their phylogenetic position and (iii) realize their utility for determining early eukaryote ecology and diversity dynamics.

### 4.1. Finding early eukaryote fossils more easily

With mudstones being the dominant repository for early eukaryote fossils [30], but also a common Proterozoic lithology [196], what makes one Proterozoic mudstone more likely to preserve early eukaryotes than another? Earlier in this article, we showed how emerging taphonomic research on Proterozoic organic-walled microfossil preservation suggests that mudstones low in TOC (<0.5 wt%) are more likely to yield fossils, offering new criteria in the search for early eukaryote fossils (figure 3*a*) [57,59]. Already the Neoproterozoic has been identified as a promising interval of geological time with its rocks bearing lower TOC than those of other intervals [57,59,80].

However, we need to find not only early eukaryote microfossils but specifically those with high fidelity preservation such that enough morphological features are preserved to precisely determine their position on the tree of life. Recognition of grades of preservation within mudstones [31,57,73] may prove important, with the highest grade fossils associated with rocks of high clay contents relative to TOC (figure 3*a*) [57] and in some cases, the binding of the clay kaolinite to fossil surfaces [62,72]. Mudstones representing palaeoenvironments where kaolinite production was favoured (tropical environments with high drainage and a low soil pH [74]) might mark fertile hunting ground for new exceptionally preserved assemblages [62].

Much remains unknown about organic-walled microfossil preservation in mudtsones, especially of the factors contributing to the highest grade material, limiting our ability to use these new mineralogical and geochemical fingerprints [57,73]. We need better characterization of the preservation of known assemblages [73]. Is there really a disparity of preservation grades? How is organic-walled microfossil preservation in Proterozoic mudstones different from the preservation of Phanerozoic palynomorphs and small carbonaceous fossils? If clay–organic interactions are important to the best Proterozoic microfossil preservation, which clay minerals are important? Do the biopolymers used by early eukaryotes matter? Microanalytical techniques, such as preparing samples with focused ion beam milling for precise chemical characterization, will likely prove promising tools for future research [62,72,197], although we should take care to determine the effects of such methods on original microfossil chemistry [198]. Geochemical experiments are also needed to help us understand what combination of pore water chemistries facilitates exceptional fossil preservation.

Important to efforts to find more early eukaryote microfossils is the advent of geochemical databases like the *Sedimentary Geochemistry and Paleoenvironments Project* (SGP) [199]. The SGP Stage 1 data product includes over 82 000 samples and over 2 700 000 individual analytical measurements [199]. This provides great scope to filter and identify samples and successions to target for new microfossil occurrences based on geochemical data indicating promising preservation potential such as TOC and clay content [73]. Although mineralogical data are currently limited in SGP [199], major element data may be indicative of mineralogical abundances [200–204].

### 4.2. Better assessing the phylogenetic position of early eukaryote fossils

A major challenge for the utility of the Proterozoic microfossil record is recognition: how do we establish the biological affinity of a fossil and its precise phylogenetic position when few morphological characters are preserved [8,10,81]? Modern eukaryote biology has seen a proliferation of molecular data to construct phylogenies, making the integration of morphological data from fossils challenging [34,120,125]. However, recently there has been a resurgence of analyses that quantitatively assess morphological disparity across large groups of modern organisms (e.g. via nonmetric multidimensional scaling—NMDS), with studies focusing on animals [205], plants (including green algae) [206] and fungi [207]. Fossil morphologies, which often include combinations of characters not found in modern taxa, can then be investigated in the context of modern morphospace. For example, Clark and colleagues showed how fossil plants commonly act as intermediates between regions of morphospace occupied by modern plant groups, providing insight into the acquisition of modern morphologies [206]. This approach has potential in terms of highlighting phylogenetically important characters that may aid recognition and in providing a quantitative framework with which to assess fossil affinity. Future research might focus on disparity of modern algae or other protist groups.

The morphological information that microfossils provide can also be increased through the use of advanced techniques. For example, the application of TEM has increased significantly over the last two decades and provides valuable ultrastructure characters of fossil cells that have aided phylogenetic interpretation [83,85,98,128,155,208]. As mentioned previously, TEM recently documented microfossil ultrastructure possibly consistent with the presence of thylakoid membranes in *Navifusa*, leading the authors to rule out a eukaryote affinity for the microfossils [98].

Fossils, however, can yield more phylogenetically informative data than what we can learn from their morphology alone. The biochemical composition of fossils is emerging as a key tool in palaeontology [209]. Materials characterization has been used to provide phylogenetic signals from preserved fossil organics and their associated elements, e.g. Raman spectroscopy [90,127,210,211], Fourier-transform infrared spectroscopy (FTIR) [127,212,213], X-ray absorption spectroscopy [83], organic mass spectrometry [144,145,214], nanoscale secondary ion mass spectrometry [215] and X-ray fluorescence [83]. Most interesting was the recent application of FTIR to distinguish microorganisms in the Devonian Rhynie Chert—an exceptionally preserved fossil assemblage from a terrestrial hot spring [212]. Preservation in chert is a taphonomic mode known to preserve Proterozoic microfossils, albeit in the marine realm [49]. Loron and colleagues resolved phylogenetic groupings of fossils from an analysis of FTIR spectra, including amoebae, animals and plants [212]. Although the distinct diagenetic conditions of individual localities will likely prevent comparisons between multiple localities, the Rhynie Chert example demonstrates the technique’s power to distinguish groups of organisms with similar morphologies within an assemblage [212]. Similarly, Sforna and colleagues provided a phylogenetic signal for the Proterozoic microfossil *A. tetragonala* by using a multiproxy approach of X-ray fluorescence and X-ray absorption spectroscopy to identify chlorophyll derivatives within its preserved cells [83].

### 4.3. Realizing the utility of early eukaryote microfossils for determining ecology and diversity dynamics

Uniquely, fossil data can provide insight into the habitat occupation of early eukaryotes. However, this aspect of their data has not been consistently explored. Few studies have looked at the palaeoecological distribution of microfossils within a single assemblage in the way of Javaux and Knoll for the Roper Group [116] or Muscente and colleagues for the Doushantuo Formation [56], and the importance of non-marine settings for the rise of eukaryotes requires further exploration [44,48,159]. Making palaeoecological reconstruction routine in microfossil studies would realize the full potential of the data that Proterozoic fossils harbour.

Efforts to produce compilations of eukaryote microfossil occurrences have highlighted the utility of fossils for documenting biodiversity dynamics across the Proterozoic [7,30,147,216]. More could be done, however, to integrate records into an accessible database of Precambrian fossils. Such a database would make fossil identification easier, with a centralized repository of fossil morphologies for new specimens to be compared against. Novel to this database might be the inclusion of samples investigated for fossils but that produced none. Such data would highlight palaeogeographic regions, intervals of geological time or lithologies that are well sampled, yet yield no fossils, perhaps indicative of lower abundance/diversity of eukaryotic life in those settings. Currently such information is not available, so there is little clarity as to the true abundance of fossil evidence.

## 5. Conclusions

The early evolution of eukaryotes marks a turning point for life on our planet. The Proterozoic fossil record has provided significant insight into this biological radiation since the recognition of its existence ~70 years ago. Today it is used in parallel with molecular clocks and biomarkers from sedimentary organic matter and together they are enabling us to reconstruct the tempo and ecology of early eukaryote evolution. However, the rarity of well-preserved fossil evidence continues to challenge these efforts. Advances in understanding the taphonomy of early eukaryotes, methods of placing them on the tree of life and recognition of the unique palaeobiological data they provide offer the prospect of a Proterozoic microfossil record with greater utility for documenting early eukaryote evolution.

## Data Availability

This article has no additional data.
